# PIK3CA Is Regulated by CUX1, Promotes Cell Growth and Metastasis in Bladder Cancer *via* Activating Epithelial-Mesenchymal Transition

**DOI:** 10.3389/fonc.2020.536072

**Published:** 2020-12-03

**Authors:** Zhongyu Wang, Jun Shang, Zhiqin Li, Huanhuan Li, Chufan Zhang, Kai He, Shikang Li, Wen Ju

**Affiliations:** ^1^Department of Urology, Union Hospital, Tongji Medical College, Huazhong University of Science and Technology, Wuhan, China; ^2^Department of Thoracic and Cardiovascular Surgery, The First Affiliated Hospital of Guangxi Medical University, Nanning, China; ^3^Department of Pharmacy, Tongji Hospital, Tongji Medical College, Huazhong University of Science and Technology, Wuhan, China; ^4^Department of Medical Oncology, Fudan University Shanghai Cancer Center, Shanghai, China

**Keywords:** PIK3CA, CUX1, EMT, prognosis, bladder cancer

## Abstract

PIK3CA is a key component of phosphatidylinositol 3-kinase (PI3K) pathway that its involvement in tumorigenesis has been revealed by previous research. However, its functions and potential mechanisms in bladder cancer are still largely undiscovered. Tissue microarray (TMA) with 66 bladder cancer patients was surveyed *via* immunohistochemistry to evaluate the level of PIK3CA and CUX1 and we found upregulation of PIK3CA in bladder cancer tissue and patients with higher level of PIK3CA presented with poorer prognosis. Overly expressed PIK3CA promoted growth, migration, invasion, and metastasis of bladder cancer cells and knockdown of PIK3CA had the opposite effect. Gain-of-function and loss-of-function studies showed that *PIK3CA* expression was facilitated by CUX1, leading to activation of epithelial-mesenchymal transition (EMT), accompanied by upregulated expression of Snail, β-catenin, Vimentin and downregulated expression of E-cadherin in the bladder cancer cell lines. Besides, over-expressed CUX1 could restore the expression of downregulated Snail, β-catenin, Vimentin and E-cadherin which was induced by PIK3CA knockdown. These results revealed that *PIK3CA* overexpression in bladder cancer was regulated by the transcription factor CUX1, and *PIK3CA* exerted its biological effects by activating EMT.

## Introduction

Bladder cancer is one of the most frequent malignancies of the urogenital system, accounted for 199,922 deaths each year. Approximately 549,393 new cases reported every year ranks 12^th^ among all types of cancer, according to 2018 Global Cancer Statistics report (1). The pathogenesis of bladder cancer is highly complex, and can be attributed to genetic factors and multiple environmental factors such as aromatic amines and tobacco (2, 3). Transitional cell carcinoma or urothelial carcinoma accounts for more than 90% of all pathological types of bladder cancer (4), there are only one-third belongs to muscle invasive bladder cancer, and the rest are characterized by a very high risk for distant metastasis (5, 6). Accumulated evidence has exhibited that genetic changes act as a vital role in the carcinogenesis and metastasis of bladder cancer, including changes in genes, such as *PIK3CA*, *NPR3*, and *FGFR3*, as well as non-coding RNAs, such as mir-93, mir-106b (7–12). Thus, exploring underlying mechanisms of bladder cancer progression in the molecular level is conducive to the investigation of corresponding targeted therapies.

PtdIns-3-kinase subunit P110-alpha (PIK3CA) is a critical regulator of carcinogenesis and metastasis (13). Various studies have ascertained that somatic mutations in *PIK3CA* can generally be found in cancers of the brain, breast, liver, and colon (14, 15). PIK3CA gene alteration and PIK3CA upregulation are frequent events for bladder cancer which promotes bladder cancer cell proliferation, invasion, and metastasis (9, 16). Previous researches have proved that the transcription factor FOXO3, can transactivate the catalytic subunit PIK3CA causing upregulated class PI3K-AKT1 activity (17). Other researches have exhibited that overly expressed miR-375 or miR-490-5p is able to decrease the expression of PIK3CA mRNA and protein, and consequently inhibit the PI3K/Akt signaling pathway (18, 19). It has been well established that PIK3CA can regulate tumor growth and progression through the PI3K pathway (13). However, more detailed analysis of the molecular mechanisms of PIK3CA-related functions in bladder cancer is still in urgent need.

In the present study, we identified Cut like homeobox1 (CUX1) as a crucial modulator of PIK3CA by mining public databases. Notably, recent researches have corroborated that the activation or overexpression of *CUX1* in tumor progression is a crucial contributor to the growth, invasion, and metastasis of tumor cells (20–24). According to literature research, this is the first study demonstrating that *PIK3CA* expression is positively correlated with *CUX1* in bladder cancer cells. In addition, epithelial-to-mesenchymal transition (EMT) is integral to cancer progression by reactivating wound healing, fibrosis and other cellular processes (25, 26). Previous studies implies that EMT may be an important factor in bladder cancer (27–29), that reduces expression of epithelial markers, such as E-cadherin, plakoglobin, and β-catenin, which have been correlated to both the grade and stage of bladder cancers (30, 31). In addition, abnormal expression of transcription factors such as Snail, Twist, Slug and ZEB1/ZEB2 has been demonstrated significant association with bladder cancer progression (32, 33).

## Materials and Methods

### Cell Lines and Culture

The human bladder cancer cell lines T24T and EJ were kindly provided by Professor Fuqing Zeng (Department of Urology, Union hospital, Tongji Medical College, Huazhong University of Science and Technology) in 2017, and subjected to DNA tests and authentication in previous studies (34, 35). Endothelial cell line HUVEC (CRL-1730) was purchased from American Type Culture Collection (ATCC, Manassas, VA, USA) in 2017. EJ and T24T cells were cultured in DMEM supplemented with 10% fetal bovine serum (FBS) (Gibco, Grand Island, NY, USA) and 1% penicillin–streptomycin (Wuhan Google Biotechnology Co., Ltd., Wuhan, China), while HUVEC cells were cultured in RPMI1640 medium. Cells were maintained at 37°C in a humidified atmosphere of 5% CO_2_.

### Bladder Cancer Tissue Microarray and Immunohistochemistry

Tissue microarray (TMA) was consisted of 56 primary bladder cancer tissues and 10 paired non-tumor tissues obtained from Shanghai Outdo Biotech Co., Ltd. Although complete histologic data were available for tissues, but only limited and incomplete clinical information were available (**Supplementary Table S1**). The protein expression of *PIK3CA* was detected by immunohistochemistry (IHC) (PR8581C, 1:1000 dilution, Thermo Fisher Scientific, USA).

### Chromatin Immunoprecipitation Assay

Chromatin immunoprecipitation (CHIP) assay was performed with EZ-CHIP kit (upstate Biotechnology) following instructions from the manufacturer with antibodies specific for *CUX1*. IgG as a negative control and histone H3 as a positive control. ABI Prism 7700 Sequence Detector was used for quantitative real-time PCR with SYBR Green PCR Master Mix (Applied Biosystems) using primer sets targeting gene promoters as shown in **Supplementary Table S2**. Chromatin immunoprecipitation assay was performed by using primary antibodies for histone H3 (ab1791, Abcam, 1:1000 dilution), IgG (ab150081, Abcam, 1:1000 dilution).

### Dual-Luciferase Reporter Assay

Human PIK3CA luciferase reporter was established by annealing complementary oligonucleotides containing four canonical CUX1 binding sites (**Supplementary Figures S3A, B**) and inserting into pGL3-Basic (Promega). The *PIKCA* promoter region is −990 to +559, and the primer for luciferase is shown in **Supplementary Table S2**. Dual-luciferase reporter assay was carried out under manufacturer’s instructions (Promega, Madison, WI).

### Western Blot

Proteins (40 μg) were collected and extracted from T24T and EJ cells, separated by gel electrophoresis, and then transferred to a PVDF membrane (Millipore, USA). Western blot analysis was performed by using primary antibodies for *PIK3CA* (PR8581C, 1:1000 dilution, Thermo Fisher Scientific, USA), *CUX1*(ab73885, 1:1000 dilution, Abcam, USA), E-cadherin (33–4000, 1:1000 dilution, Thermo Fisher Scientific, USA), Snail (PA1-86737, 1:1000 dilution, Thermo Fisher, USA), β-catenin (AB_2533982, 1:1000 dilution, Thermo Fisher, USA), Vimentin (AB_2544707, 1:1000 dilution, Thermo Fisher, USA), and β-actin (PA1-183-HRP, 1:2000 dilution, Thermo Fisher Scientific, USA); followed by goat anti-rabbit HRP-labeled secondary antibody (AB_2556786, 1:500 dilution, Thermo Fisher Scientific, USA).

### Plasmids and shRNA

To construct *PIK3CA* and *CUX1* overexpression plasmids, human *PIK3CA* and *CUX1* cDNA were synthesized by TSINGKE (Wuhan, China), then inserted into pcDNA3.1 (Geenseed Biotech Co., Guangzhou, China). Short hairpin RNAs targeting human *PIK3CA* and *CUX1* (**Supplementary Table S3**) were cloned into GV298 (Geenseed Biotech Co., Guangzhou, China). Lipofectamine 2000 (Life Technologies) was used for transfection according to the manufacturer’s instructions. The transfected cells were selected with G418 (Life Technologies) for 4 to 6 weeks to generate stable cell lines.

### RT-PCR and Real-Time Quantitative RT-PCR

Total RNA was isolated using the RNeasy Mini Kit (Qiagen Inc., Valencia, CA). Reverse transcription reactions were conducted with Transcriptor First Strand cDNA Synthesis Kit (Roche, Indianapolis, IN). The PCR primers were designed using Premier Primer 5.0 software, and the primers for *PIK3CA*, *CUX1*, *Snail, β-catenin, vimentin, E-cadherin*, and *β-actin* are listed in **Supplementary Table S2**. Real-time PCR was performed with SYBR Green PCR Master Mix (Applied Biosystems, Foster City, CA). The fluorescent signals were collected during the extension phase, *C*_t_ values of the samples were calculated, and the transcript levels were analyzed using the 2^−ΔΔ^*^C^*^t^ method.

### Cell Viability Assay

Cell Counting Kit-8 (CCK8) was purchased from Trans Gen Biotech, China. The treated cells were plated into 96-well plates at a density of 4 × 10^3^ cells per well with 100 μl DMEM containing 10% fetal bovine serum and cultured at 37°C in a 5% CO_2_ humidified incubator. After 48 h, 10 μl of CCK-8 (Solarbo, China) was added to each well, and then the cells were incubated at 37°C for 4 h. The absorbance at 450 nm was measured using a micro-plate reader (Eon, BioTeck, USA). All experiments were done with 6–8 wells per experimental condition and repeated at least three times.

### Wound Healing Assay

T24T and EJ cells were cultured in six-well plates and the cell layer was scratched with the fine end of 200 μl pipette tips (time, 0 h). Plates were washed twice with phosphate buffer saline to remove detached cells, and incubated with complete growth medium. Cell migration was photographed using10 high-power fields, at 0, 24, and 36 h after scratching. Remodeling was measured as reduction in the width of the scratch, normalized to the 0 h control, and expressed as relative migration (μm).

### Cell Invasion Assay

Matrigel invasion assay was performed using membranes coated with Matrigel matrix (Invitrogen Incorporated, USA). Homogeneous single cell suspensions (1×10^5^ cells/well) were added to the upper chambers and allowed to invade for 24 h at 37°C in a 5% CO_2_ incubator. The invasion rate was quantified by counting the invading cells in at least three random fields.

### Colony Formation Assay

Treated T24T and EJ Bladder cancer cells (3 × 10^2^) were seeded in six-well plates and allowed to grow at 37°C in a 5% CO_2_ incubator, and observed every 24 to 48 h. The medium was replenished regularly and cells were allowed to grow for 4 weeks. Cells were then fixed and stained with methylene blue or crystal violet, photomicrographs were captured, and colonies were counted.

### Tube Formation Assay

Polymerization of 50 µl of growth factor-reduced Matrigel was polymerized in 96-well plates. HUVECs were starved for 24 h in incomplete RPMI1640 medium, suspended in tumor cell pretreated RPMI1640 medium, and then and 5 × 10^4^ cells/well were added to Matrigel-coated wells, and incubated at 37°C for 18 h. Evaluation of angiogenic activity was calculated by measuring the length of the wall formed between discrete endothelial cells in each well of the control.

### An Orthotopic Model of Murine Bladder Cancer

The 24G detaining needle, paraffin oil, and disinfectants required for the experiment were provided by the department of Urology, Wuhan Union Hospital. All animal experiments were approved by the Animal Care Committee of Tongji Medical College (approval number: 2016–0057). For the *in vivo* tumor growth studies, 4-week-old female BALB/c nude mice were randomly divided into two groups (n = 5 for each group). T24T cells stably transfected with sh-PIK3CA plasmids or control vector were prepared at a concentration of 1 × 10^6^ cells/100 μl. Nude mice were anesthetized with 100 μl 10% chloral hydrate by intraperitoneal injection, and a transurethral indwelling catheter was placed using a 24G needle. The bladder was drained of urine, and then 100 μl 0.1 mmol/l HCL was injected into the bladder *via* the catheter, maintained for 15 s, and then followed by injection of 100 μl 0.1 mmol/l NAOH. Bladders were rinsed twice with sterile normal saline and 100 μl of T24T cells at a concentration of 1 × 10^6^ cells/ml were then injected into the bladder *via* the catheter using a 1 ml syringe. The syringes were attached to the catheters to prevent efflux of the cell suspensions, and attachment was maintained for at least 5 min to allow the cells to attach to the burn sites. Nude mice were housed in a specific pathogen-free environment for 2 weeks and regularly monitored.

### *In Vivo* Imaging of Small Animals

The *in vivo* Optical Imaging System (FX PRO, Alameda, CA) was used to acquire fluorescent images of orthotopic models of bladder cancer and lung metastases in nude mice. Before imaging, mice were anesthetized with 10% chloral hydrate by intraperitoneal injection. Red fluorescence was detected using 570 and 600 nm filter sets for excitation and emission, respectively. The Xenogen Living Image Software (Bruker MI SE. Alameda, CA) was used for collecting signals. Statistical analysis was performed using Origin8.1 (OriginLab, Northampton, MA) software. The bladder tissues and the lung samples were removed and prepared for histological examination (IHC or H&E staining).

### Statistical Analysis

All analyses were completed using GraphPad Prism 7 or SPSS 20.0 software, with *P* < 0.05 considered statistically significant. Two-tailed unpaired *t*-test was used to evaluate statistical significance between the mean values of the two groups. Correlation analysis was assessed by Chi-square test and Pearson’s correlation test. Chi-square test was used to compare the difference in the proportion of high and low expression of PIK3CA in bladder cancer of different clinical stages.

## Results

### PIK3CA Was Expressed at High Levels in Bladder Cancer Tissues and Positively Associated With Poor Overall Survival Time of Bladder Cancer Cases

**Figure 1:**
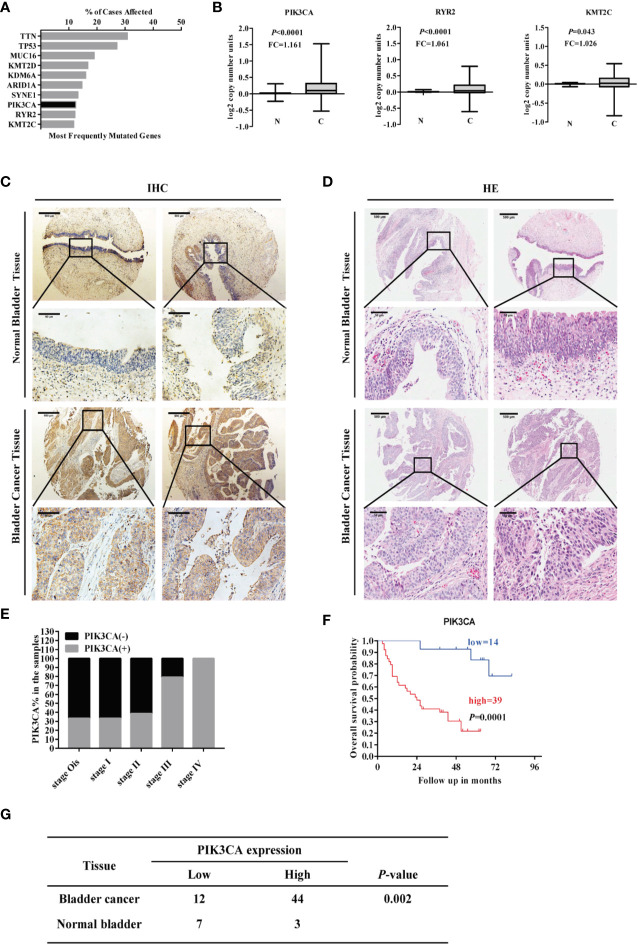
*PIK3CA* was highly expressed in bladder cancer tissues and positively correlated with poor overall survival time of patients with bladder cancer. **(A)** Top ten potential mutated genes in bladder cancer by mining TCGA database on December 01, 2018. **(B)** The RNA-sequence of the top ten mutated genes with statistical significance by mining TCGA database (PIK3CA, *P*<0.0001; RYR2, *P*<0.0001; KMT2C, *P*=0.043). **(C, D)** Representative immunohistochemical and H&E staining images of PIK3CA expression in bladder cancer tissues and adjacent normal bladder tissues on a TMA (magnification ×40 and × 400). Scale bars represent 500 μm (low magnification) and 50 μm (high magnification). **(E)** The expression of PIK3CA in different clinical stages of bladder cancer. **(F)** The survival curve of bladder cancer patients with low or high expression of PIK3CA (*P*=0.0001). **(G)** The amount of bladder cancer group and Normal control group with the low and high expression of PIK3CA respectively in TMA (*P*=0.002).

Mining the publicly available The Cancer Genome Atlas (TCGA) database (https://cancergenome.nih.gov/) showed the top ten most commonly mutated genes (*KMT2C*, *RYR2*, *PIK3CA*, *SYNE1*, *ARID1A*, *KDM6A*, *KMT2D*, *MUC16*, *TP53*, *TTN*) ranked by the percent of cases affected (**Figure 1A**). Among the top ten genes, PIK3CA, RYR2, and KMT2C were highly expressed in the bladder cancer compared to normal bladder tissue (*P* < 0.001) (**Figure 1B**). The following seven genes showed no statistical significance: *SYNE1*, *ARID1A*, *KDM6A*, *KMT2D*, *MUC16*, *TP53*, *TTN* (**Supplementary Figure S1**). IHC assay indicated that PIK3CA was located in the cytoplasm of bladder cancer cells (**Figure 1C**), and displayed that PIK3CA protein was detected in 44/56 (78.6%) bladder cancer cases and with a higher positive rate than normal cases (*P* = 0.002). H&E staining of the TMA (**Figure 1D**) can also be exhibited (**Figure 1G**). PIK3CA immunoreactivity was notably higher in older patients with bladder cancer (*P* = 0.045), with more advanced clinical stage (*P* = 0.016), and higher rate positive lymph node metastasis (*P* = 0.031) (**Figure 1E** and **Supplementary Table S1**). Finally, Kaplan–Meier survival curves of 53 cases of bladder cancer patients derived from the TMA showed that patients with high expression of PIK3CA (*n* = 39) exhibited significantly worse survival as compared to those with low expression (*n* = 14) (*P* < 0.001) (**Figure 1F**). All the above findings indicate that PIK3CA is highly expressed in bladder cancer and positively related with poor clinical outcome. They have provided strong evidence that IPK3CA stands as a key component in the process of bladder cancer development.

### PIK3CA Promoted the Proliferation, Migration, Invasion, and Angiogenesis of Bladder Cancer *In Vitro*

**Figure 2:**
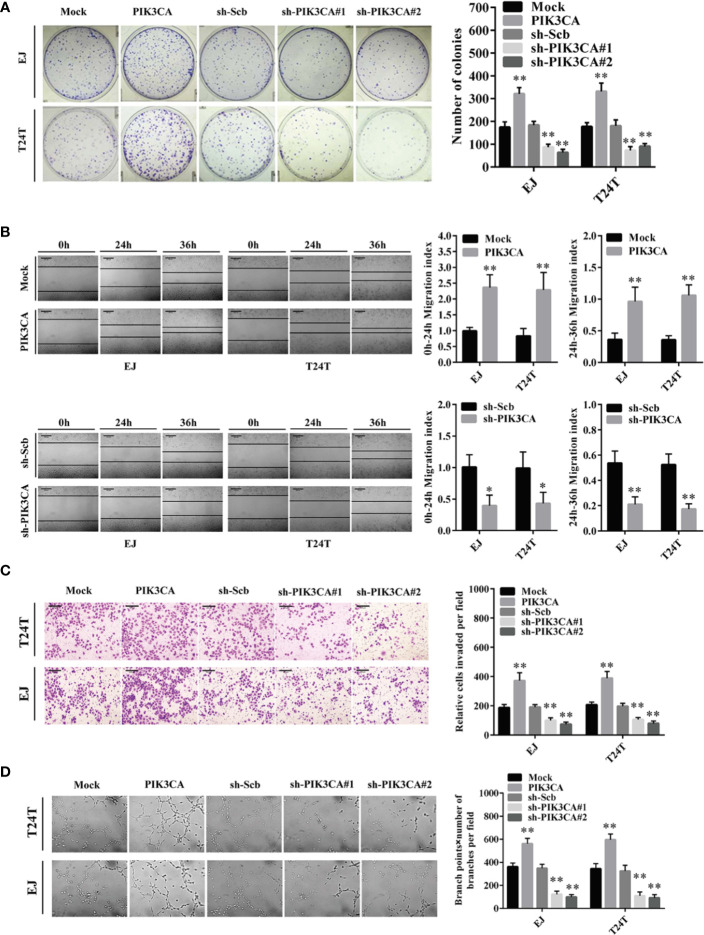
PIK3CA promoted the growth, migration, invasion and angiogenesis of bladder cancer cells *in vitro*. **(A)** Cell proliferation was detected by colony formation assay in EJ and T24T cells. **(B)** The effect of *PIK3CA* on cell migration was evaluated by wound healing assay in T24T and EJ cells. Scale bar indicates 200 μm. **(C)** Cell invasion of T24T and EJ cells was tested by transwell invasion assay. Scale bar indicates 100 μm. **(D)** Representation (left) and quantification (right) of tube formation assay showing the angiogenic capability of bladder cancer cells. Data are mean ± SEM, *n* = 3. *P < 0.05, **P < 0.01 (Student’s *t*-test).

To further probe the potential effects of *PIK3CA* in bladder cancer cells, we first transfected bladder cancer cell lines EJ and T24T with a *PIK3CA*-expressing vector and verified the expression of PIK3CA by qRT-PCR and Western blot (**Supplementary Figures S2A, B**). In CCK8 and colony formation assays, overexpression or knockdown of *PIK3CA* resulted in increased or decreased viability and growth of EJ and T24T cells, in comparison to control group (mock or sh-Scb) (**Figure 2A** and **Supplementary Figures S3C, D**). In a scratch assay, overexpression of *PIK3CA* promoted the migratory behaviors of EJ and T24T cells, meanwhile, knockdown of *PIK3CA* showed the opposite effect (**Figure 2B**). Transwell analysis demonstrated that PIK3CA up-regulation or knockdown made cells more invasive or less invasive, respectively (**Figure 2C**). Additionally, tube formation assay indicated similar effects on angiogenesis in EJ and T24T cells (**Figure 2D**).

In conclusion, these findings indicated that the tumor promoting roles of *PIK3CA* through regulatory effects on proliferation, migration, invasion, and angiogenesis of bladder cancer cells.

### The Transcription Levels of *PIK3CA* Was Increased by CUX1 Regulation

**Figure 3:**
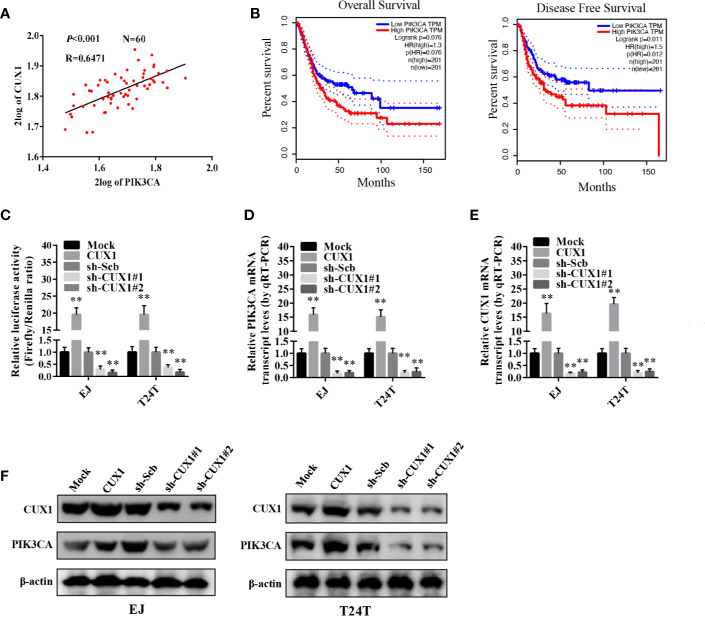
The transcription of *PIK3CA* was increased by CUX1 through direct binding to the *PIK3CA* promoter. **(A)** The relationship between *PIK3CA* and *CUX1* was assessed by using the Genomics Analysis and Visualization Platform R2 Database (*P*<0.001). **(B)** The over survival and disease free survival curve of bladder cancer patients with low or high expression of *PIK3CA* (OS, P = 0.076, DFS, P = 0.012). **(C)** Dual-luciferase assay showing the activity of the PIK3CA promoter in bladder cancer cells stably transfected with empty vector (mock), *CUX1*, sh-Scb, and sh-*CUX1*. **(D–F)** The transcript and protein levels of *PIK3CA* were detected using Real-time PCR and Western blot, respectively in bladder cancer cells stably transfected with an empty vector, *CUX1*, sh-Scb, and sh-*CUX1*. **P < 0.01.

We used the R2 database to analyze the correlation between the transcription factor collection and PIK3CA, and found that CUX1 and PIK3CA have a significant correlation, so CUX1 and PIK3CA were further explored. To verify the hypothesis that CUX1 may affect the expression of *PIK3CA* in bladder cancer, we performed computational assessment by transcription factor binding site analysis through the UCSC Database (https://genome.ucsc.edu/index.html) and Genomatix software (36). There are four potential binding sites of CUX1 located in the PIK3CA promoter (**Supplementary Figures S3A, B**). There was a positive correlation between PIK3CA and CUX1expression (P < 0.001) based on R2 Database (Tumor Bladder-Orntoft-60-MAS5.0-U133A, http://hgserver1.amc.nl/cgi-bin/r2/main.cgi) (**Figure 3A**). The results of 404 TCGA data also suggest that there may be a positive correlation between PIK3CA and CUX1 (P < 0.001) (**Supplementary Figure S4A**). Using TCGA bladder cancer data to perform survival analysis and disease free survival analysis on PIK3CA and CUX1, we found that the expression of PIK3CA is closely related to disease free survival, but the expression of CUX1 is not significantly related to patient survival and disease free survival. (**Figure 3B**, **Supplementary Figure S4B**). Trying to reveal the function of *CUX1* in bladder cancer cells, we first transfected cell lines EJ and T24T with a *CUX1*-ovexpressing vector and verified the expression of CUX1 by qRT-PCR and Western blot (**Supplementary Figures S4C, D**). ChIP assay indicated that binding to the *PIK3CA* promoter could be increased or decreased with overexpression or knockdown of *CUX1* in bladder cancer cells, respectively. Moreover, dual-luciferase reporter assay demonstrated that up-regulation of CUX1 facilitated the transcription activity of *PIK3CA* in bladder cancer cells (**Figure 3C**). To further identify the effect of CUX1 on the expression of *PIK3CA* in bladder cancer cell lines, we performed qRT-PCR and Western blot assay with overexpression or silencing of *CUX1*. We found that over-expression of CUX1 elevated mRNA and protein level of PIK3CA and CUX1 in EJ and T24T cells compared to control group (mock or sh-Scb). Additionally, knockdown of *CUX1* induced decrease in mRNA and protein of PIK3CA and CUX1in EJ and T24T cells compared to control group (mock or sh-Scb) (**Figures 3D–F**). Together, the above results demonstrated that CUX1 stimulated transcription activity *via* direct interaction with the binding site of PIK3CA promoter.

### Overexpression of *PIK3CA* Restored the Proliferation, Migration, Invasion, and Angiogenesis of Bladder Cancer Cells Through Knockdown of *CUX1*

**Figure 4:**
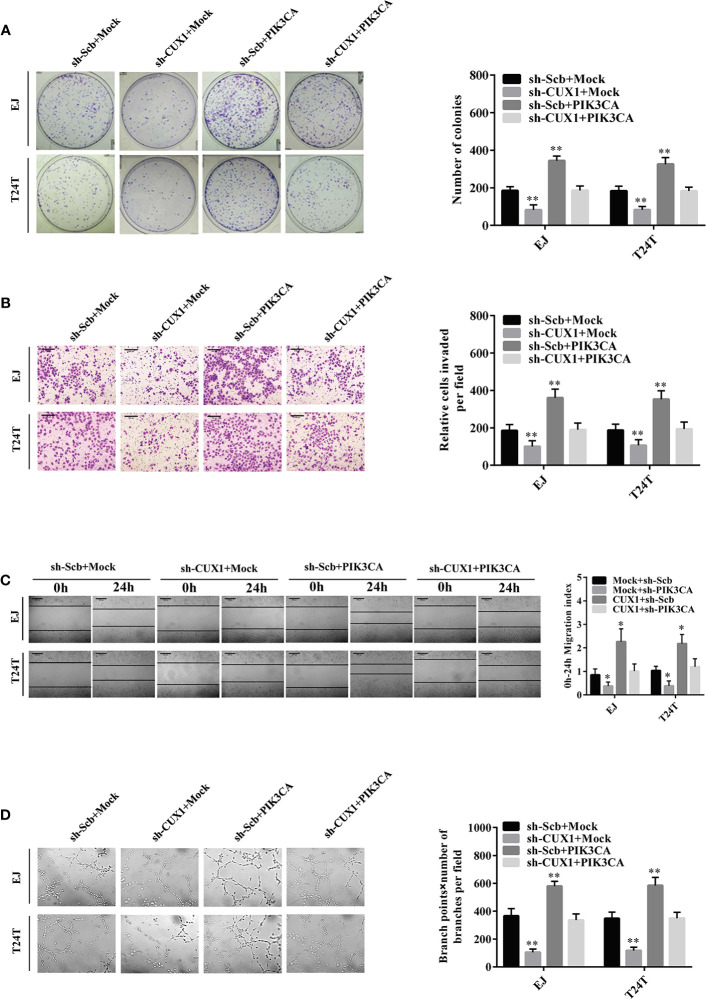
Overexpression of *PIK3CA* restored the growth, migration, invasion, and angiogenesis of bladder cancer cells through knockdown of *CUX1*. **(A–D)** Colony formation, transwell migration, Matrigel invasion, and tube formation assays were used to investigate the proliferation, migration, invasion, and angiogenic capacity, respectively, of bladder cancer cells stably transfected with mock, *CUX1*, sh-Scb, and sh-*CUX1*, and those co-transfected with *PIK3CA* and sh-*PIK3CA*. Data are mean ± SEM, *n* = 3. *P < 0.05, **P < 0.01 (Student’s *t*-test).

Given that PIK3CA has been reported participating in the proceeding of tumor proliferation, migration, and invasion (37, 38), and in combination with the evidence that the expression of *PIK3CA* can be directly regulated by CUX1, the effects of *CUX1* knockdown and *PIK3CA* restoration on bladder cancer was further explored. Colony formation assay and CCK8 assay indicated suppressed anchorage-independent growth of EJ and T24T cells after CUX1 knock down in comparison to control group stably transfected with an empty vector (mock) (**Figure 4A** and **Supplementary Figure S5F**). Restoration of *PIK3CA* overexpression reversed the inhibitory effect on bladder cancer cell growth *in vitro* (**Figure 4A** and **Supplementary Figure S5F**). Transwell analysis showed that *CUX1* knockdown attenuated invasion ability of EJ and T24T cells (**Figure 4B**). In a scratch assay, *CUX1* knockdown attenuated migration ability of EJ and T24T cells (**Figure 4C**). Tube formation assay showed that cancer cells treated with medium preconditioned by *CUX1*-knockdown, significantly suppressed angiogenesis capabilities (**Figure 4D**). Additionally, under the condition of CUX knock down, restoration of PIK3CA overexpression showed revived migration, invasion and angiogenesis ability in EJ and T24T cells. (**Figures 4B–D**). It revealed same outcome like above that, *PIK3CA* knockdown weaken the proliferation, migration, invasion and angiogenesis capabilities of EJ and T24T cells, however restoration of *CUX1* overexpression rescued bladder cancer cells from their defects resulted from knockdown of *CUX1* (**Supplementary Figures S5A–E**). Our results confirmed a vital regulatory role of CUX1 in PIK3CA-induced aggressiveness and angiogenesis of bladder cancer cells

### PIK3CA Promote Bladder Cancer Progression by Activating EMT Related Makers—Snail, E-cadherin, Vimentin, and β-Catenin

**Figure 5:**
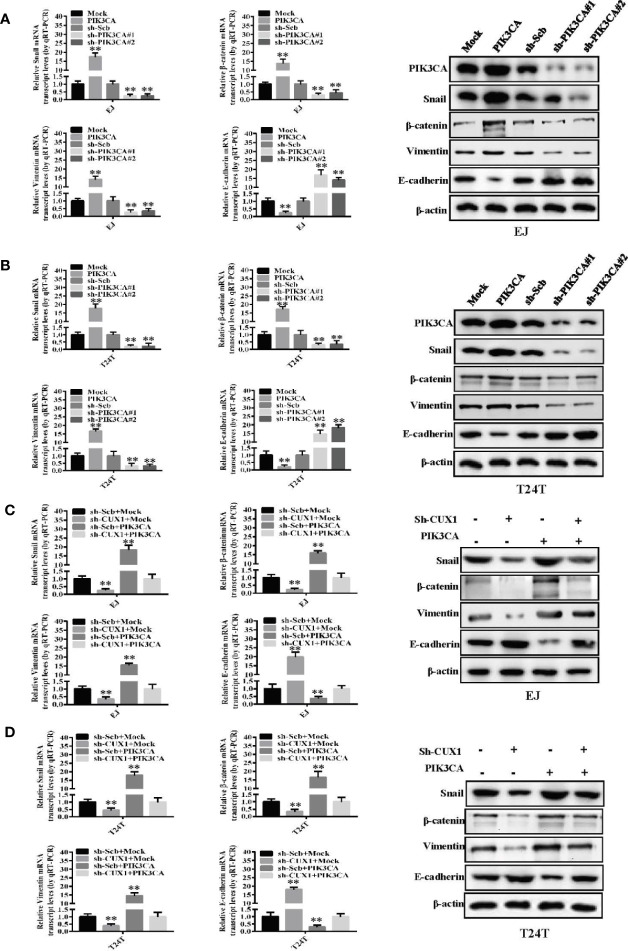
PIK3CA promoted bladder cancer progression by activating EMT related makers—Snail, β-catenin, vimentin and E-cadherin. **(A, B)** The mRNA expression and protein levels of Snail, β-catenin, vimentin and E-cadherin were determined using RT-PCR and Western blot, respectively, in EJ or T24T cells stably transfected with mock, *PIK3CA*, sh-Scb and sh-*PIK3CA*. **(C, D)** The transcript and protein levels of Snail, β-catenin, vimentin and E-cadherin in EJ or T24T cells stably transfected with mock, *CUX1*, sh-Scb, and sh-*CUX1*, and those co-transfected with *PIK3CA* and sh-*PIK3CA*, as detected by quantitative real-time PCR and Western blot. **P < 0.01.

Although the functional role of PIK3CA in EMT has been investigated in several cancers (39), there are only a few reports in bladder cancer demonstrating involvement of PIK3CA in the process of EMT. Therefore, to investigate this potential functional relationship, we evaluated the expression of key EMT-related markers—Snail, β-catenin, Vimentin and E-cadherin using western blot and qRT-PCR. In EJ cells and T24T cells, over-expression of PIK3CA could upregulate the expression of Snail, β-catenin and Vimentin, while downregulate the expression of E-cadherin significantly (**Figures 5A, B**) Consistently, knock down of PIK3CA caused downregulation of Snail, β-catenin, and Vimentin, while E-cadherin was upregulated significantly (**Figures 5A, B**). In agreement with the results of a previous study (20), Western blot and qRT-PCR demonstrated that knocking down CUX1 also led to downregulation of Snail, β-catenin, and Vimentin, while upregulated the expression of E-cadherin (**Figures 5C, D**). In accordance with our expectation, over-expressed of PIK3CA could restore the expression level of Snail, β-catenin, Vimentin, and E-cadherin which decreased by CUX1 knockdown (**Figures 5C, D**). Meanwhile, we also found that knocking down PIK3CA resulted in down-regulation of Snail, β-catenin, and Vimentin, while up-regulation of E-cadherin (**Supplementary Figures S6A, B**). As expected, over-expressed of CUX1 could restore the expression level change of Snail, β-catenin, Vimentin, and E-cadherin which was induced by PIK3CA knockdown (**Supplementary Figures S6A, B**). These findings suggest that PIK3CA was targeted by CUX1 and the activation of CUX1/PIK3CA axis and consequently regulation of EMT pathway may contribute to promote bladder cancer cell progression.

### Knockdown of PIK3CA Suppressed the Growth and Metastasis of Bladder Cancer Cells *In Vivo*

**Figure 6:**
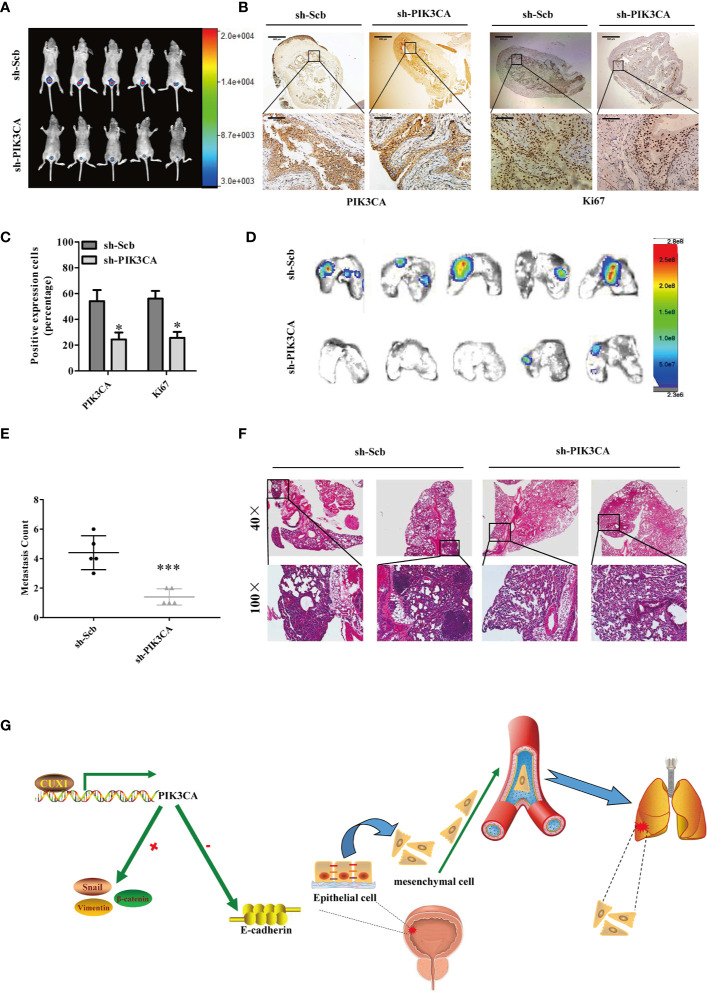
Knockdown of *PIK3CA* suppressed the growth and metastasis of bladder cancer cells *in vivo*. **(A)** Fluorescence intensity of sh-*PIK3CA* or negative control orthotopic bladder tumors in BALB/c nude mice. **(B)** Immunohistochemical staining of the expression of *PIK3CA* and Ki67 in bladder specimens. Original magnification, ×40 and ×400. Scale bars represent 500 μm (low magnification) and 50 μm (high magnification). **(C)** The positive expression of Ki67 and *PIK3CA* per microscopic field of the sh-Scb or sh-*PIK3CA* group. **(D–F)** Non-invasive bioluminescence imaging and H&E staining of the lung tissues were shown by *in vivo* tail vein metastatic assay. Original magnification, ×40 and ×400. Scale bars represent 500 μm (low magnification) and 50 μm (high magnification). **(G)** Schematic model of CUX1-PIK3CA-EMT oncoprotein axis is shown. CUX1 contributes to the upregulation of *PIK3CA* in bladder cancer by promoting transcriptional activity of *PIK3CA*. PIK3CA further activates EMT, thereby generating a CUX1-PIK3CA-EMT regulatory circuit to promote bladder cancer invasion and metastasis. Data are mean ± SEM, *n* = 3. *P < 0.05, ***P < 0.001 (Student’s *t-*test).

Next, we explored the effect of *PIK3CA* knockdown on tumor growth and metastasis *in vivo*. T24T cells treated with sh-*PIK3CA* were implanted into the bladders of BABL/C nude mice. Downregulated PIK3CA mice group (transfected with sh-PIK3CA) had a stronger fluorescence intensity detected in the bladder area, which showed that downregulated PIK3CA mice had a lower tumor cells than sh-Scb mice (transfected with an empty vector) (**Figure 6A**). Compared with sh-Scb mice tumor issue, the ratio of tumor cell with positive Ki67(proliferation related biomarker) was lower in sh-PIK3CA mice (**Figures 6B, C**). Consistent with the results of Ki67, sh-PIK3CA mice tumor issue had lower PIK3CA positive tumor cells than sh-Scb mice. The metastasis model was established by T24T cells stably transfected with sh-*PIK3CA*, showed that sh-*PIK3CA* group had fewer lung metastatic colonies than the sh-Scb group (**Figures 6D, F**). These findings proved that the bladder tumor growth, migration, invasion, and angiogenesis was substantially inhibited by knocking down *PIK3CA in vitro*.

## Discussion

PIK3CA, located at chromosome 3q26.3 encodes a 120-kDa protein, was first detected through *in situ* hybridization in 1994 by Volinia et al. (40–42). Former research has revealed that abnormally increased PIK3CA contributes to the growth and invasion of multiple cancer cell line, including breast, ovarian, colorectal, and gastric cancers (14, 15). In addition, the amplification of PIK3CA is a common event in many kinds of human tumors (43–45). Many studies have shown that the onset and progression of bladder cancer are related to the PIK3CA gene, and its mutations have been detected in in bladder cancer cases with different stages and grades (46, 47). In the current study, we revealed that PIK3CA is overexpressed in bladder cancer tissues, and PIK3CA participates in the proliferation, migration, and invasion of bladder cancer cells. It has been demonstrated that the primary molecular mechanisms regulating PIK3CA overexpression in cancer were mutation and amplification (43). Recently, studies have indicated that the PI3K/AKT/mTOR pathway may be commonly activated in multiple human cancers by molecular abnormalities such as PIK3CA mutations (48, 49). Furthermore, transcription factors such as FOXO3a can affect the expression of PIK3CA in CML (50).

Transcription factors (TF) play significant roles in carcinogenesis and cancer progression in diverse types of cancer, such as breast cancer (51), prostate cancer (52), pancreatic cancer (53), and other cancers (54). However, the detailed mechanism of TF function in regulating PIK3CA in bladder cancer is complex and not well understood. Using publicly available databases, we identified four potential CUX1 binding sites in the promoter region of PIK3CA. Interestingly, there are no literature reports of *PIK3CA* regulation by CUX1 in bladder cancer. CUX1 belongs to the homeodomain transcription factor family, And its involvement in the proliferation and progression of several cancers has been proved (55–57). Recently, studies have reported that the overexpression and activation of *CUX1* may have a crucial impact on promoting cancer cell growth, invasion, and metastasis (20–22, 58, 59). CUX1 has been described as a transcriptional activator as well as a repressor, but its cancer-promoting effects have garnered increased research interest (57, 58). However, the potential mechanism of CUX1 regulation of *PIK3CA* in bladder cancer cells requires further investigation. In the current study, we revealed the positive correlation between *CUX1* and *PIK3CA* expression in bladder cancer tumor samples and cell lines. Significantly, restoration of *PIK3CA* expression rescued bladder cancer cells from CUX1-knockdown inhibition of growth, aggressiveness, and angiogenesis. This suggests that CUX1 may exert its tumor promoting function through transcriptional regulation of *PIK3CA* in bladder cancer. The Ripka study has demonstrated that CUX1 expression was induced by activation of Akt/protein kinase B signaling, and decreased by PI3K inhibitors in pancreatic cancer (24). In contrast, our study claimed that the expression of PIK3CA is regulated by CUX1 in bladder cancer. Considering the inter-tumor heterogeneity between tumors, there are differences of genotypic and phenotypic in different types of tumors cells. Even in the same type of tumor, there is intra-tumor heterogeneity, that is, there are differences of genotypic and phenotypic in tumor cells. In addition, heterogeneity also exists between tumor microenvironment, between tumor cells and tumor circulating cells, and between different tumor cells in the same tumor tissue. Based on the above study, we think that in different tumor types, even the same signal pathway may be regulated by different genes or molecules. In the field of tumor research, the mutual regulation between the two molecules is common, so even our research is different from others (24), which precisely illustrates the complexity of the tumor itself and the tumor microenvironment. Of course, we will further explore the relationship between CUX and PIK3CA in bladder cancer. In summary, we determined that CUX1 is a vital TF of *PIK3CA*, regulating its expression and resulting changes in cellular functions in bladder cancer cells.

Epithelial-to-mesenchymal transition (EMT) is a biologic process that transforms epithelial cells into a mesenchymal cell phenotype (25). During EMT, cell junction proteins are downregulated, apical-basal polarity is lost and several biochemical changes will occur. All the processes stated above will allow the cells to migrate, invade, resist apoptosis, and remodel the extracellular matrix (ECM) (26, 60, 61). In recent years, mounting evidence has shown that cell migration and invasion are closely related to EMT, thereby promoting cancer cells to erode through surrounding tissues and translocate to distant tissues (62, 63). Previous studies have shown that malat1 promotes bladder cancer invasion and metastasis by activating TGF-β–induced EMT (64). In addition, DAB2IP can also be a down-stream target of miR-92b in bladder cancer cells to facilitate EMT and promote tumor cell migration or invasion (65). Additionally, activation of EMT is related to many factors, including an increase in the expression of Snail, and the decrease or loss of E-cadherin (66–68). In our study, we found activation of *PIK3CA* resulted in an increase in the expression of Snail and a decrease in the expression of E-cadherin at the mRNA and protein level, while inhibition of *PIK3CA* had the opposite effect. In our research, although we found that over-expression of PIK3CA could upregulate the expression of Snail, β-catenin, and Vimentin, while downregulate the expression of E-cadherin significantly (**Figures 5A, B**). However, the simple use of molecular markers to judge the occurrence of EMT in cells driven by PIK3CA requires further demonstration. With the continuous deepening of EMT research, EMT has been updated from the initial binary state to a process of continuous dynamic change (69). At the same time, epithelial–mesenchymal plasticity provides us with ideas for further understanding of tumor metastasis (70). Therefore, we need to pay attention to the specific EMT state when discussing EMT-related mechanisms. In addition, when exploring the influence mechanism of genes on EMT in cells, we need to explore the E/M phenotype of cells in depth.

Here, we propose a model of CUX1-PIK3CA-EMT oncoprotein axis, to illustrate how PIK3CA is activated and contributes to bladder cancer progression and metastasis (**Figure 6G**). It is entirely possible that PIK3CA may also be involved in the progression of bladder cancer through other regulatory pathways. Therefore, more studies are required to further investigate how PIK3CA act in bladder cancer development and progression. In addition, our study provides a possible new therapeutic target for the exploration of novel therapy of bladder cancer.

Our study showed that PIK3CA is up-regulation in bladder cancer and promoted the proliferation, migration, invasion and angiogenesis of bladder cancer *in vitro*. Considering that amplification and mutations of PIK3CA is a common event in human tumors, and PIK3CA mutations and/or PTEN loss cooperates with TP53 mutation to drive the development of bladder cancer (71). PTEN has a potential impact on response to mTOR inhibitors *via* the PI3K/Akt/mTOR pathway in the therapy of bladder cancer (72). Seront et al. document that PTEN loss was associated with resistance to the mTOR inhibitor in patients with advanced bladder cancer (73) Western blot and qRT-PCR.

The inhibition of PI3K/Akt can sensitize PTEN-deficient tumor cells to the effects of mTOR inhibition. In our study, whether the regulation of PIK3CA by CUX1 involves the loss of PTEN is worthy of further study. We will collect the tumor samples from patients with bladder cancer and the relationship between loss of PTEN and the mutation of PIK3CA can be observed by “Next-generation” sequencing technology (NGS) sequencing, laying a foundation for the treatment of bladder cancer.

In summary, our study demonstrates for the first time that *PIK3CA* is overexpressed in bladder cancer, and is regulated by the transcription factor CUX1. PIK3CA functions to promote proliferation and metastasis of bladder cancer by activating EMT. Furthermore, the expression of *PIK3CA* is an independent favorable prognostic factor for bladder cancer patients. Thus, as an oncogene, *PIK3CA* may serve as a potential target for the diagnosis and treatment of bladder cancer.

There are still many flaws in our research that need to be resolved. For example, the conclusion that CUX1 stimulates the expression of PIK3CA requires more experiments to further demonstrate. At the same time, our current findings suggest that PIK3CA may be related to EMT in regulating the occurrence and development of bladder cancer, but how PIK3CA regulates the dynamic process of EMT requires further research in the future.

## Data Availability Statement

The data that support the findings of this study are available from the corresponding authors upon reasonable request.

## Ethics Statement

The animal study was reviewed and approved by the Animal Care Committee of Tongji Medical College (approval number: 2016-0057).

## Author Contributions

ZW conceived of the study and performed the experiments. ZW, JS, and CZ wrote the manuscript. JS and ZW analyzed the data and mined the database. WJ and SL initiated, organized, and supervised the study. ZL revised the manuscript. HL and KH performed animal experiments. All authors contributed to the article and approved the submitted version.

## Funding

This work was supported by a grant from the National Natural Science Foundation of China (NSFC81660488). Bladder cancer cells were provided by Pro. Fuqing Zeng.

## Conflict of Interest

The authors declare that the research was conducted in the absence of any commercial or financial relationships that could be construed as a potential conflict of interest.

## Supplementary Material

Supplementary Figure 1 RNA-sequence of genes in TCGA database. The RNA-sequence of the top ten mutated genes with no statistically significant by mining TCGA database (TTN, *P*=0.93; TP53, *P*=1.000; MUC16, *P*=0.985; KMT2D, *P*=0.070; KDM6A, ARID1A, *P*=0.804; SYNE1, *P*=1.000).

Supplementary Figure 2 The exploration of the potential effects of PIK3CA in bladder cancer cells. (a) and (b) Screening the potential effects of PIK3CA in bladder cancer cells *via* RT-PCR and Western blot by transfection of bladder cancer cell lines with sh-Scb and sh-*PIK3CA* plasmids.

Supplementary Figure 3 PIK3CA promoted the growth of bladder cancer cells *in vitro*. (a) and (b) Scheme of the potential binding site of *CUX1* within the *PIK3CA* promoter. (c) and (d) CCK-8 assay showing the proliferation of EJ and T24T cells stably transfected with a PIK3CA overexpressing or silencing plasmid.

Supplementary Figure 4 Screening the potential effects of *CUX1* in bladder cancer cells. (a) Analysis of 404 bladder cancers in the TCGA database suggests that PIK3CA and CUX1 are significantly positively correlated. (b) In the TCGA 402 bladder cancer data, the expression of CUX1 has no obvious correlation with patient over survival and disease free survival. (c) and (d) Screening the potential effects of CUX1 in bladder cancer cells *via* RT-PCR and Western blot by transfection of bladder cancer cell lines with sh-Scb and sh-*CUX1* plasmids.

Supplementary Figure 5 Overexpression of *CUX1* restored the growth, migration, invasion, and angiogenesis of bladder cancer cells through knockdown of *PIK3CA*. (a)–(d) Colony formation, transwell migration, Matrigel invasion, and tube formation assays were used to investigate the proliferation, migration, invasion, and angiogenic capacity, respectively, of bladder cancer cells stably transfected with mock, *CUX1*, sh-Scb, and sh-CUX1, and those co-transfected with *PIK3CA* and sh-PIK3CA.Data are mean ±SEM, *n* = 3. **P* < 0.05, ***P* < 0.01, ****P* < 0.001 (Student’s *t*-test). (e) The proliferation of EJ and T24T cells stably transfected with mock, *PIK3CA*, sh-Scb, and sh-*PIK3CA*, and those co-transfected with *CUX1* and sh-*CUX1* as detected by CCK-8 assay. (f) The proliferation of EJ and T24T cells stably transfected with mock, *CUX1*, sh-Scb, and sh-*CUX1*, and those co-transfected with *PIK3CA* and sh-*PIK3CA* as detected by CCK-8 assay.

Supplementary Figure 6 Overexpression of *CUX1* restored EMT-related makers. (a) and (b) The transcript and protein levels of Snail, and E-cadherin in EJ or T24T cells stably transfected with mock, *PIK3CA*, sh-Scb, and sh-*PIK3CA*, and those co-transfected with *CUX1* and sh-*CUX1* as detected by quantitative real-time PCR.

## Abbreviations

IK3CA, PtdIns-3-kinase subunit P110-Alpha; CUX1, cut like homeobox 1; EMT, epithelial–mesenchymal transition; siRNA, small interfering; TMA, tissue microarray; ChIP, chromatin immunoprecipitation; RT-PCR, reverse transcription PCR; qRT-PCR, real-time polymerase chain reaction; IHC, immunohistochemistry; IF, immunofluorescence.
