# Supplementary figures and images for: PIK3CA Is Regulated by CUX1, Promotes Cell Growth and Metastasis in Bladder Cancer via Activating Epithelial-Mesenchymal Transition
Source: Front Oncol. 2020 Dec 3;10:536072. doi: 10.3389/fonc.2020.536072 (PMC7744743; doi:10.3389/fonc.2020.536072)

Supplementary Figure S1

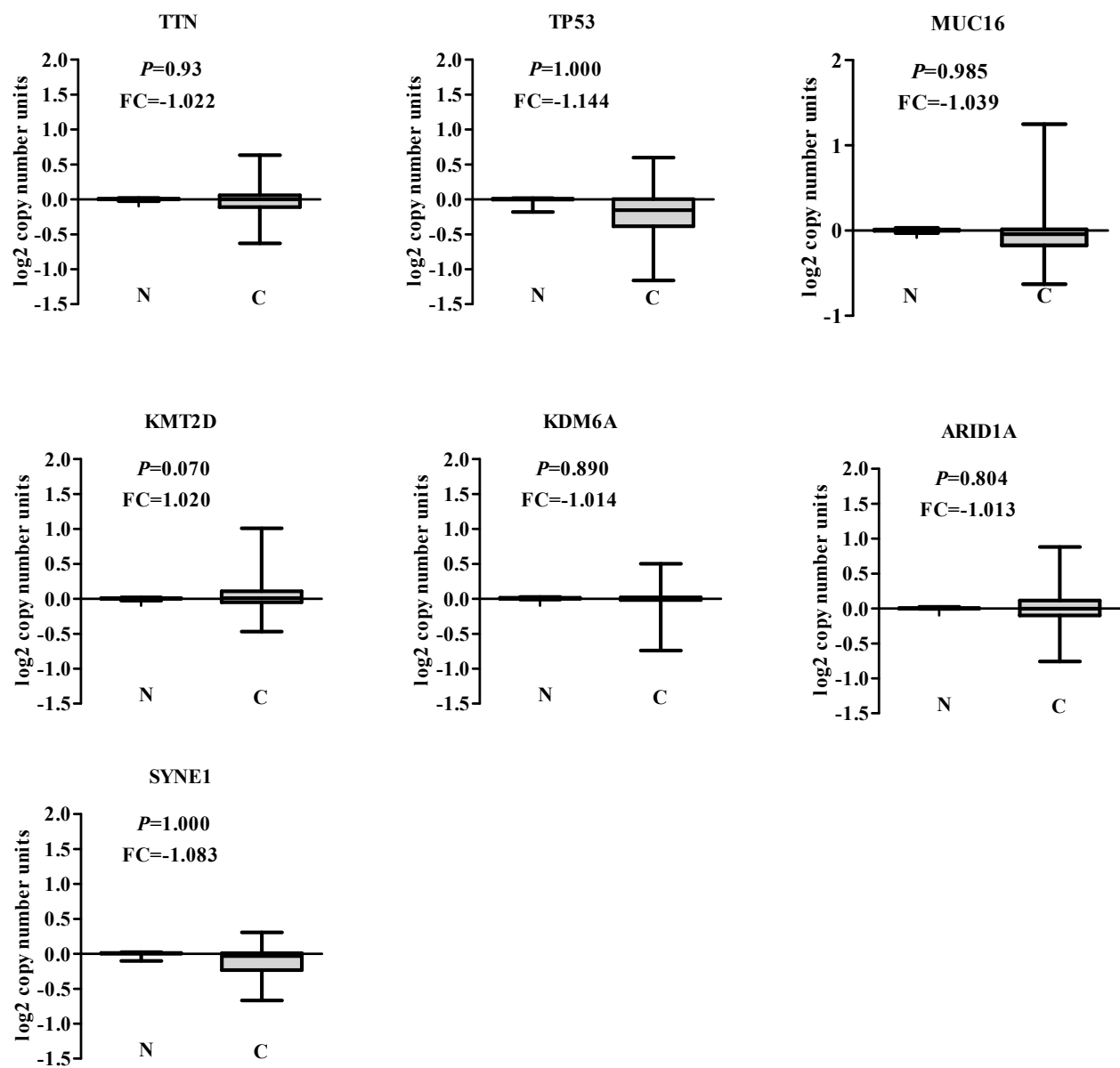

Supplement: Supplementary Figure 1 — RNA-sequence of genes in TCGA database. The RNA-sequence of the top ten mutated genes with no statistically significant by mining TCGA database (TTN, P=0.93; TP53, P=1.000; MUC16, P=0.985; KMT2D, P=0.070; KDM6A, ARID1A, P=0.804; SYNE1, P=1.000). [file Image_1.pdf]

Supplementary Figure S2

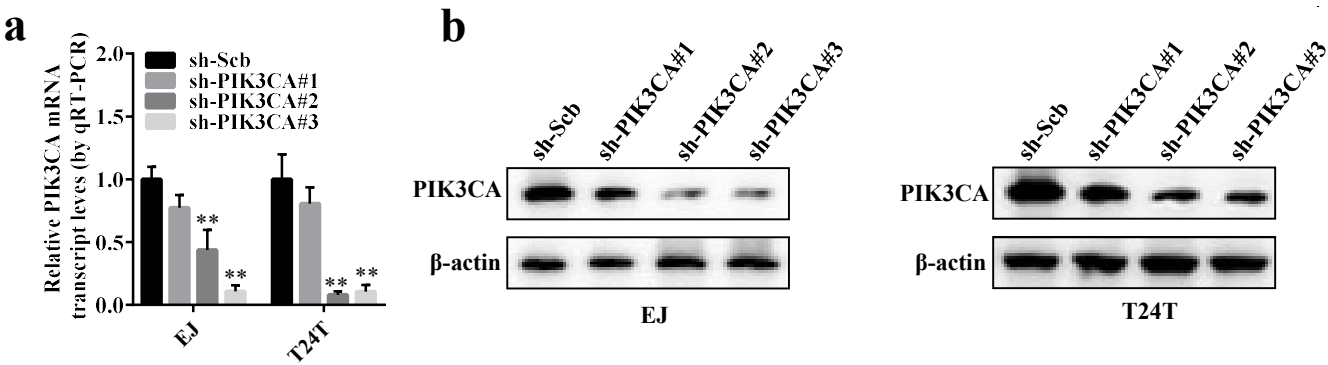

Supplement: Supplementary Figure 2 — The exploration of the potential effects of PIK3CA in bladder cancer cells. (a) and (b) Screening the potential effects of PIK3CA in bladder cancer cells via RT-PCR and Western blot by transfection of bladder cancer cell lines with sh-Scb and sh-PIK3CA plasmids. [file Image_2.pdf]

Supplementary Figure S3

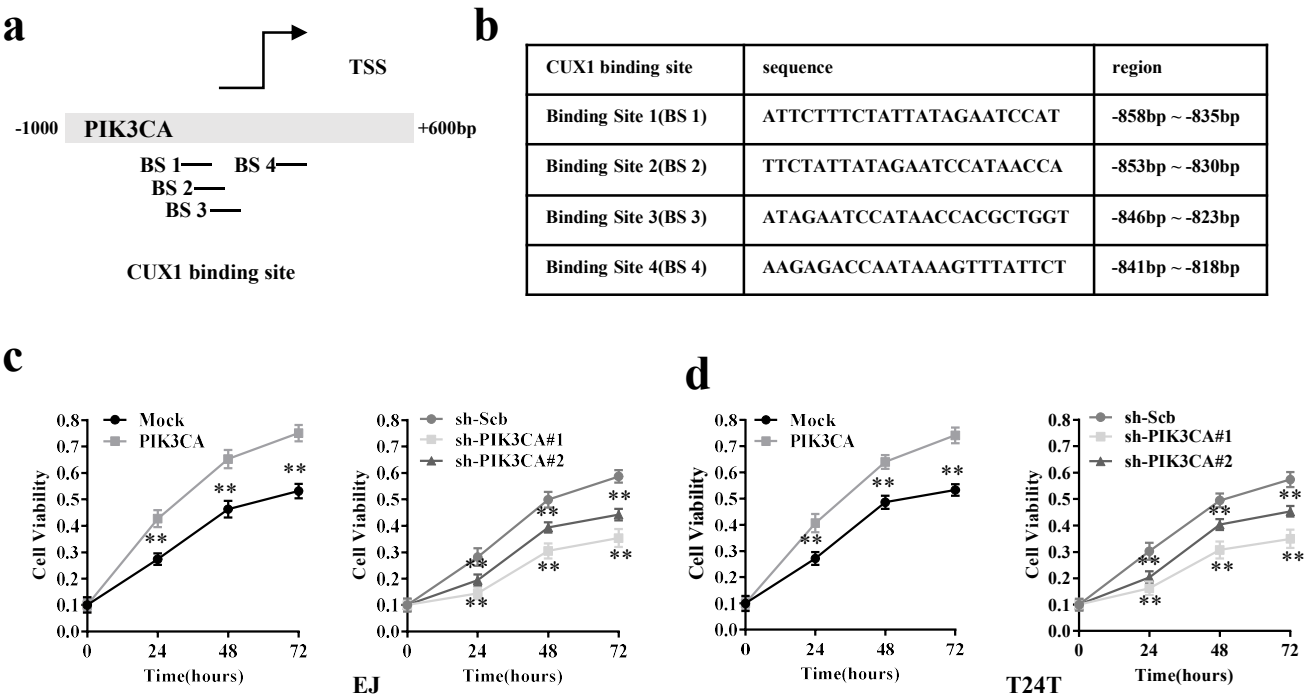

Supplement: Supplementary Figure 3 — PIK3CA promoted the growth of bladder cancer cells in vitro. (a) and (b) Scheme of the potential binding site of CUX1 within the PIK3CA promoter. (c) and (d) CCK-8 assay showing the proliferation of EJ and T24T cells stably transfected with a PIK3CA overexpressing or silencing plasmid. [file Image_3.pdf]

Supplementary Figure S4

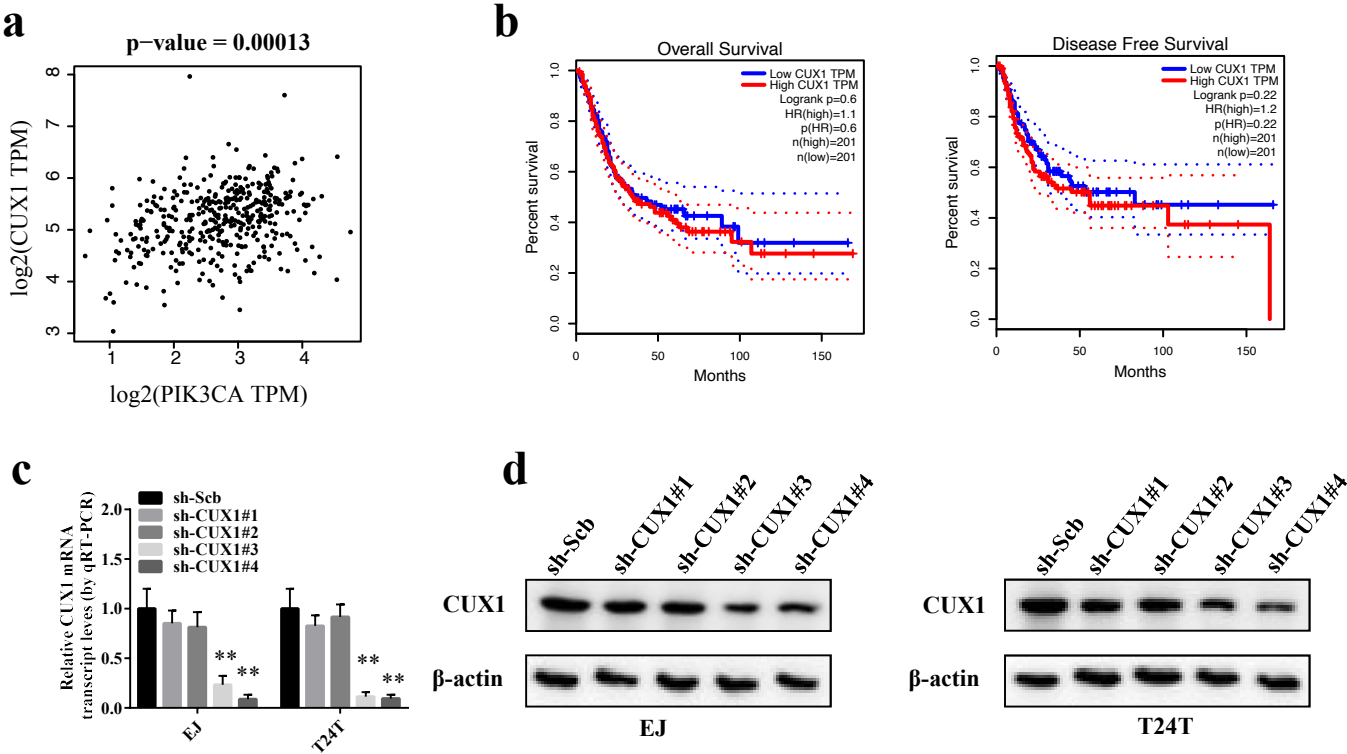

Supplement: Supplementary Figure 4 — Screening the potential effects of CUX1 in bladder cancer cells. (a) Analysis of 404 bladder cancers in the TCGA database suggests that PIK3CA and CUX1 are significantly positively correlated. (b) In the TCGA 402 bladder cancer data, the expression of CUX1 has no obvious correlation with patient over survival and disease free survival. (c) and (d) Screening the potential effects of CUX1 in bladder cancer cells via RT-PCR and Western blot by transfection of bladder cancer cell lines with sh-Scb and sh-CUX1 plasmids. [file Image_4.pdf]

Supplementary Figure S5

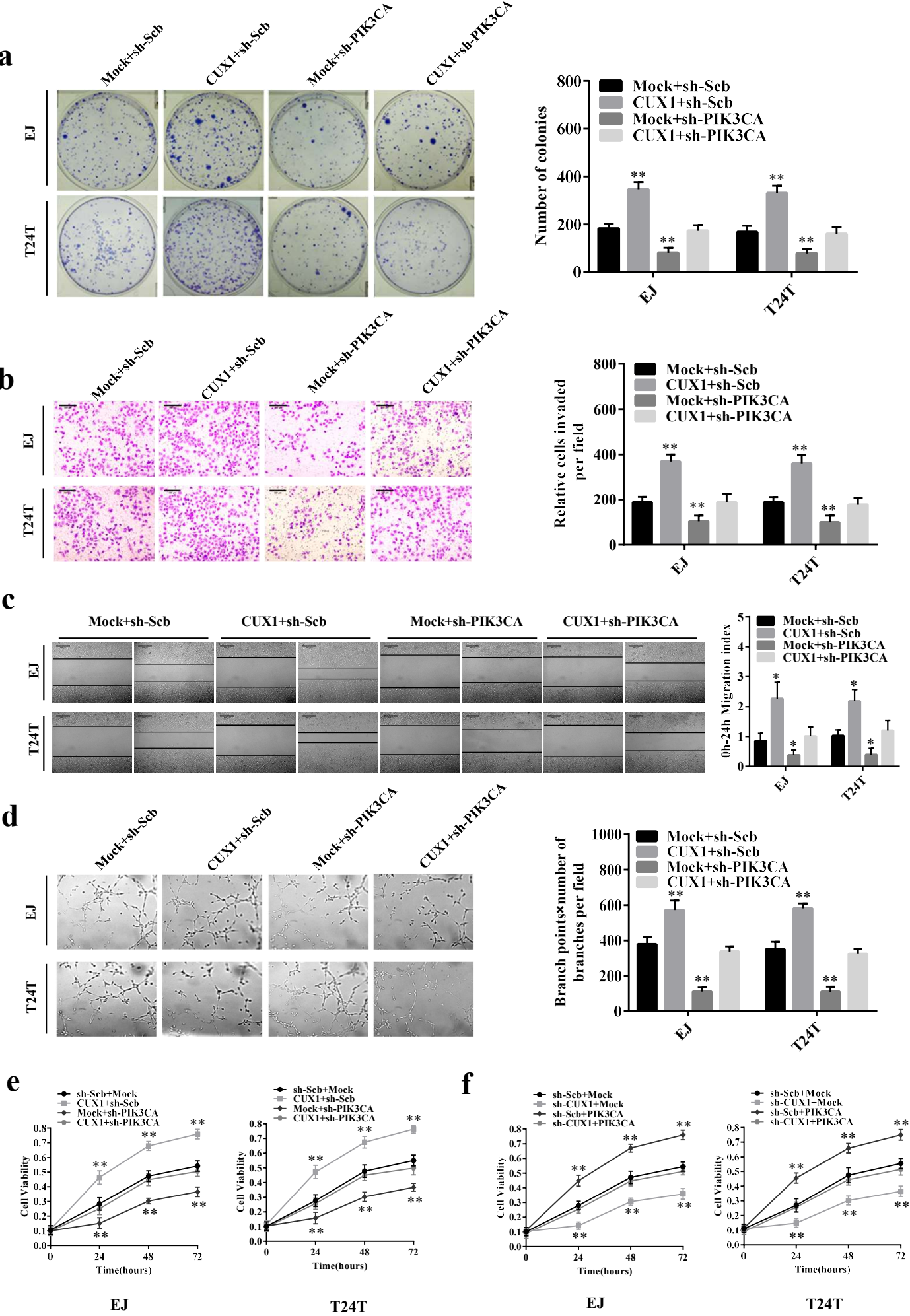

Supplement: Supplementary Figure 5 — Overexpression of CUX1 restored the growth, migration, invasion, and angiogenesis of bladder cancer cells through knockdown of PIK3CA. (a)–(d) Colony formation, transwell migration, Matrigel invasion, and tube formation assays were used to investigate the proliferation, migration, invasion, and angiogenic capacity, respectively, of bladder cancer cells stably transfected with mock, CUX1, sh-Scb, and sh-CUX1, and those co-transfected with PIK3CA and sh-PIK3CA.Data are mean ±SEM, n = 3. *P < 0.05, **P < 0.01, ***P < 0.001 (Student’s t-test). (e) The proliferation of EJ and T24T cells stably transfected with mock, PIK3CA, sh-Scb, and sh-PIK3CA, and those co-transfected with CUX1 and sh-CUX1 as detected by CCK-8 assay. (f) The proliferation of EJ and T24T cells stably transfected with mock, CUX1, sh-Scb, and sh-CUX1, and those co-transfected with PIK3CA and sh-PIK3CA as detected by CCK-8 assay. [file Image_5.pdf]

Supplementary Figure S6

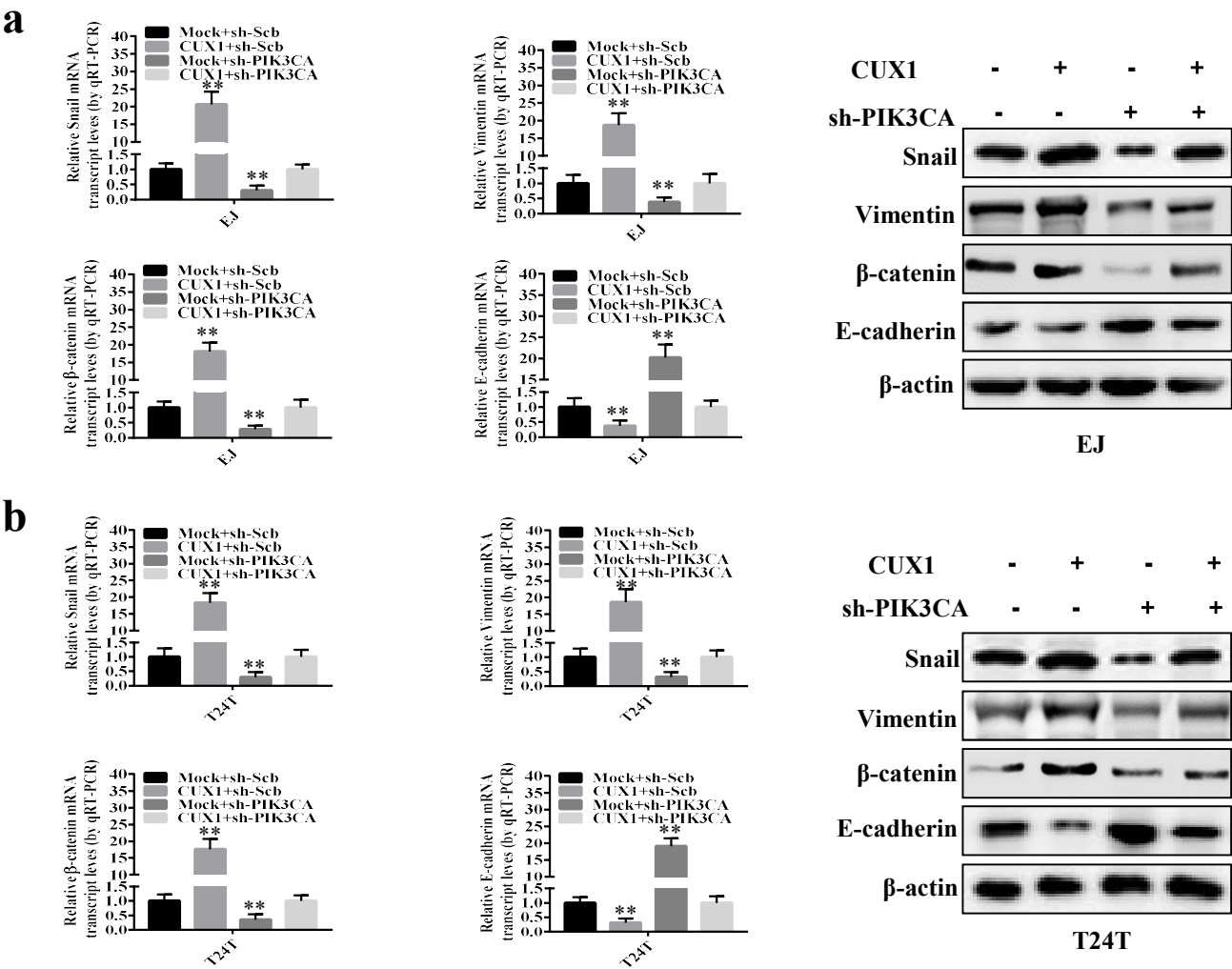

Supplement: Supplementary Figure 6 — Overexpression of CUX1 restored EMT-related makers. (a) and (b) The transcript and protein levels of Snail, and E-cadherin in EJ or T24T cells stably transfected with mock, PIK3CA, sh-Scb, and sh-PIK3CA, and those co-transfected with CUX1 and sh-CUX1 as detected by quantitative real-time PCR. [file Image_6.pdf]
