# Supplementary material for: PIK3CA Is Regulated by CUX1, Promotes Cell Growth and Metastasis in Bladder Cancer via Activating Epithelial-Mesenchymal Transition
Source: Front Oncol. 2020 Dec 3;10:536072. doi: 10.3389/fonc.2020.536072 (PMC7744743; doi:10.3389/fonc.2020.536072)
Supplement: Supplementary file 7 [file Table_1.docx]

| **Clinicopathologic factor** | **Total** | **PIK3CA expression** | ***P*-value** |
| --- | --- | --- | --- |
|  | **n（%）** | **n（%）** |  |
| **Age (years)** |  |  |  |
| **≤65** | **19（38.8）** | **16（84.2）** | **0.045** |
| **>65** | **30（61.2）** | **17（56.7）** |  |
| **Sex** |  |  |  |
| **Male** | **41（83.7）** | **27（65.9）** | **0.614** |
| **Female** | **8（16.3）** | **6（75.0）** |  |
| **Size (diameter)** |  |  |  |
| **≤5 cm** | **33（67.3）** | **21（63.6）** | **0.426** |
| **>5cm** | **16（32.7）** | **12（75）** |  |
| **Bladder wall invasion** |  |  |  |
| **Tis/T1** | **9（18.4）** | **3（33.3）** | **0.016** |
| **T2/T3/T4** | **40（81.6）** | **30（75.0）** |  |
| **Lymph node metastasis** |  |  |  |
| **Negative** | **41（83.7）** | **25（61.0）** | **0.031** |
| **Positive** | **8（16.3）** | **8（100）** |  |
| **Distant metastasis** |  |  |  |
| **Negative** | **19（100）** | **33（67.3）** |  |
| **Positive** | **0** | **0** |  |
| **Clinical Stage** |  |  |  |
| **Ois/I** | **9（18.4）** | **3（33.3）** | **0.016** |
| **II/III/IV** | **40（81.6）** | **30（75.0）** |  |

**Supplementary Table S1**

**PIK3CA expression in Bladder Caner by TMA**
