# Supplementary material for: PIK3CA Is Regulated by CUX1, Promotes Cell Growth and Metastasis in Bladder Cancer via Activating Epithelial-Mesenchymal Transition
Source: Front Oncol. 2020 Dec 3;10:536072. doi: 10.3389/fonc.2020.536072 (PMC7744743; doi:10.3389/fonc.2020.536072)
Supplement: Supplementary file 8 [file Table_2.docx]

**Supplementary Table S2**

**Primer sets used for Luciferase, qRT-PCR , and ChIP**

| **Primer set** | **Primers** | | **Sequence** | **Product size (bp)** | **Application** |
| --- | --- | --- | --- | --- | --- |
| PIK3CA-pGL3.0 | Forward | 5’-GGCATAGCAAAAGGTCTCCACG-3’ | | 1550 | Luciferase |
|  | Reverse | 5’-CATACCCCTGCCGGGCGATTTA-3’ | |  |  |
| PIK3CA | Forward | 5’-TGTCTCCTCTAAACCCTG-3’ | | 174 | qRT-PCR |
|  | Reverse | 5’-CTTGCCGTAAATCATCCC-3’ | |  |  |
| CUTL1 | Forward | 5'-ACCCGTCACCAAAAACCATCG-3' | | 131 | qRT-PCR |
|  | Reverse | 5'-CCCGGCCTGAATTTCCTCAAT-3' | |  |  |
| Snail | Forward | 5’-CTGGGTGCCCTCAAGATG-3’ | | 261 | qRT-PCR |
|  | Reverse | 5’-GTGGAGCAGGGACATTCG-3’ | |  |  |
| E-cadherin | Forward | 5’-AAAGCCTCAGGTCATAAACA-3’ | | 120 | qRT-PCR |
|  | Reverse | 5’-TGGGTTGGGTCGTTGTAC-3’ | |  |  |
| β-catenin | Forward | 5'-AAAGCGGCTGTTAGTCACTGG-3' | | 215 | qRT-PCR |
|  | Reverse | 5'-CGAGTCATTGCATACTGTCCAT-3' | |  |  |
| vimentin | Forward | 5'-GACGCCATCAACACCGAGTT-3' | | 238 | qRT-PCR |
|  | Reverse | 5'-CTTTGTCGTTGGTTAGCTGGT-3' | |  |  |
| β-actin | Forward | 5'-TGCCCATCTACGAGGGGTATG-3' | | 156 | qRT-PCR |
|  | Reverse | 5'-TCTCCTTAATGTCACGCACGATTT-3' | |  |  |
| PIK3CA-CUTL1-CHIP | Forward | 5’-AGAAGAACGCACAGCAACG-3’ | | 143 | CHIP |
|  | Reverse | 5’-TTACACCCATAGAGGAAACGA-3’ | |  |  |
