# Supplementary material for: PIK3CA Is Regulated by CUX1, Promotes Cell Growth and Metastasis in Bladder Cancer via Activating Epithelial-Mesenchymal Transition
Source: Front Oncol. 2020 Dec 3;10:536072. doi: 10.3389/fonc.2020.536072 (PMC7744743; doi:10.3389/fonc.2020.536072)
Supplement: Supplementary file 9 [file Table_3.docx]

**Supplementary Table S3   Oligonucleotide sets used for short hairpin RNAs**

| shRNA | shRNA Target Sequence | Manufacturer |
| --- | --- | --- |
| sh-PIK3CA#1 | 5′-GGACCTCAATTCACCTCATAG-3′ | Genechem Co., LTD., Shanghai |
| sh-PIK3CA#2 | 5′-GCAACCTACGTGAATGTAAAT-3′ | Genechem Co., LTD., Shanghai |
| sh-PIK3CA#3 | 5′-GCTAGAGACAATGAATTAAGG-3′ | Genechem Co., LTD., Shanghai |
| sh-CUTL1#1 | 5′- AAGAAGAACACTCCAGAGGATTT-3′ | Genechem Co., LTD., Shanghai |
| sh-CUTL1#2 | 5′- AAGAATCTTCTCGTTTGAAACTT-3′ | Genechem Co., LTD., Shanghai |
| sh-CUTL1#3 | 5′-GGAGCCAGGTTGAAGAGAGAA-3′ | Genechem Co., LTD., Shanghai |
| sh-CUTL1#4 | 5′-GAGCCAGGTTGAAGAGAGAAC -3′ | Genechem Co., LTD., Shanghai |
